# AIDS-Related Non-Hodgkin's Lymphoma in the Era of Highly Active Antiretroviral Therapy

**DOI:** 10.1155/2012/485943

**Published:** 2012-02-06

**Authors:** Prakash Vishnu, David M. Aboulafia

**Affiliations:** ^1^Floyd & Delores Jones Cancer Institute, Virginia Mason Medical Center, Seattle, WA 98101, USA; ^2^Division of Hematology-Oncology, University of Washington, Seattle, WA 98195, USA

## Abstract

In economically developed countries, AIDS-related lymphoma (ARL) accounts for a large proportion of malignances in HIV-infected individuals. Since the introduction of highly active anti-retroviral therapy (HAART) in 1996, epidemiology and prognosis of ARL have changed. While there is a slight increase in the incidence of Hodgkin's lymphoma in HIV-infected individuals, use of HAART has contributed to a decline in the incidence of non-Hodgkin's lymphoma (NHL) and also a decrease in the overall incidence of ARL. Strategies that employ HAART, improved supportive care, and the use of Rituximab with multi-agent chemotherapy have contributed to improved rates of complete remission and survival of patients with ARL that rival those seen in stage and histology matched HIV negative NHL patients. Most recent clinical trials demonstrate better outcomes with the use of rituximab in ARL. Tumor histogenesis (germinal center vs. non-germinal center origin) is associated with lymphoma-specific outcomes in the setting of AIDS-related diffuse-large B cell lymphoma. High-dose chemotherapy (HDCT) and autologous stem cell rescue (ASCT) can be effective for a subset of patients with relapsed ARL. HIV sero-status alone should not preclude consideration of ASCT in the setting of ARL relapse. Clinical trials investigating the role of allogeneic hematopoietic stem cell transplant in ARL are currently underway.

## 1. Introduction

Non-Hodgkin's lymphoma (NHL) has been associated with human immunodeficiency virus (HIV) infections since the beginning of the acquired immune deficiency syndrome (AIDS) epidemic. The initial case definition of AIDS by the US Centers for Disease Control and Prevention (CDC) in 1982 included AIDS-defining malignancies such as Kaposi's sarcoma and primary central nervous system lymphoma (PCNSL), with subsequent inclusion of peripheral intermediate and high-grade B-cell NHL [1]. People living with HIV/AIDS (PLWA) are also at a significantly greater risk of developing Hodgkin's disease compared to the general population but this has not yet been added to the CDC case definition of AIDS [2]. 

HIV seropositivity increases the risk of developing NHL by 60–165-fold [3, 4]. AIDS-related lymphomas (ARLs) tend to present with high-grade B-cell histology, advanced-stage disease, and an aggressive clinical course. Prior to the advent of highly active antiretroviral therapy (HAART) in 1996, ARL was associated with a dismal prognosis, particularly in those patients who had compromised performance status, advanced immune dysfunction, and limited hematopoietic reserve. With the introduction of HAART, the survival of patients with ARL has improved substantially and appears to be comparable to that of their HIV-negative NHL counterparts. Advances in chemotherapy regimens, antiretroviral drugs, and supportive care have led to more aggressive management of ARL compared to the pre-HAART era. Several ARL chemotherapy trials have incorporated the anti-CD20 monoclonal antibody rituximab with multiagent chemotherapy; however, optimal therapy of ARL is still not clearly defined [5–12]. 

In this paper, we highlight several current strategies for treatment of AIDS-related non-Hodgkin's lymphoma including specific systemic chemo-biologic therapies. We also briefly review various prognostic tools and factors which influence therapeutic outcomes and potential for treatment-related toxicities. We do so through a systematic review of peer-reviewed publications identified through searches of MEDLINE/PubMed from July 2005 to June 2011. The ongoing phase II and phase III trials for ARL were searched from the US National Institute of Health's web resource, http://clinicaltrials.gov/, a registry of clinical trials conducted in the United States and worldwide. Keywords were used alone and with the modifiers of *treatment, novel therapies, AIDS-related lymphoma, prognostic and biomarkers, *and* HIV/AIDS*. Bibliographies from these references were reviewed. Criteria used for study selection included study design, English language, and relevance to clinicians.

## 2. Pathobiology of ARL

ARL are comprised of a narrow spectrum of histologic types consisting almost exclusively of aggressive B-cell tumors, derived from either germinal centers or postgerminal centers. While majority of tumors are diffuse large B-cell lymphomas (DLBCLs), a few ARL are B-cell immunoblastic lymphomas and Burkitt's or Burkitt's-like small noncleaved lymphoma. Even more rare are PCNSLs. The World Health Organization has classified HIV-related lymphomas into three categories: lymphomas also occurring in immunocompetent patients, those specifically occurring in HIV-positive patients, and those occurring in other immunodeficiency states (Table 1) [13].

HIV creates a milieu of combined immune suppression and chronic antigenic stimulation in lymph nodes [14]. This environment with dysregulated cytokine release and impaired dendritic function, along with presence of concomitant infection (e.g., Epstein Barr Virus [EBV], Human Herpes Virus type 8 (HHV-8), and cytomegalovirus) may promote a permissive environment for HIV-induced polyclonal B-cell expansion and impaired T-cell immunosurveillance, culminating in lympho-proliferative disorders [15]. 

The cytokine profile of HIV-infected individuals at risk for ARL demonstrates increased markers of B-cell activation such as interleukin- (IL-) 6, IL-10, and soluble CD-30 compared to HIV-positive controls who do not develop ARL [16]. Although the molecular mechanisms responsible for B-cell transformation associated with ARL have not been completely elucidated, many different ARL-associated molecular lesions have been described, including chromosome translocation and activation of the C*-MYC* oncogene, inactivation of p53 tumor-suppressor gene, somatic mutations in Bcl-6, and overexpression of EBV oncoprotein 4 (e.g., latency membrane proteins 1 and 2). These chromosome breaks and/or molecular lesions likely have significant downstream effects which lead to impaired lymphocyte differentiation and cell-cycle control [15, 17]. Recently, preclinical studies and genome-wide DNA profiling of ARL demonstrated that B-cell-receptor-related signaling is frequently disrupted in DLBCL tumor tissues of PLWA compared to DLBCL in immunocompetent patients. This suggests HIV-associated B-cell dysregulation, and aberrant tumor-specific intracellular signaling may be important in promoting a subset of ARLs [18, 19]. Use of HAART has been associated with a significant reduction in ARL risk [4, 20, 21]. HAART may modify lymphomagenic stimuli in several ways, such as improving responsiveness of EBV-specific cytotoxic T-cell lymphocytes, increasing EBV-specific antibodies, and stabilizing immune perturbations that may contribute to B-cell proliferation [19, 22]. 

## 3. Prognostic Factors for AIDS-Related Lymphoma in the Pre-HAART and HAART Era

Early in the AIDS epidemic the clinical course of ARL was dominated by advanced stage disease, concomitant and life-threatening opportunistic infections (OIs), and poor response to treatment. Efforts to treat patients with ARL using aggressive and complex chemotherapy regimens led to unacceptable toxicity and early death while low-dose chemotherapy regimens yielded modest benefit—only 10% of patients survived for 2 years [6]. Various treatments led to dismal outcomes with the majority of patients succumbing to advancing NHL or superimposed OIs [7, 23–25]. Adverse prognostic factors for ARL included a CD4+ count of less than 100 cells/*μ*L, age older than 35, history of injection drug use, poor performance status, elevated serum lactate dehydrogenase (LDH), advanced stage of disease, and prior AIDS diagnosis [26]. Expectations for survival were dismal with a median overall survival (OS) of 46 weeks in “good prognosis” patients with a score of 0 or 1 and just 18 weeks in “poor prognosis” patients with a score of 3 or 4.

Control of HIV viral replication through HAART has emerged as a major positive prognostic factor for patients with ARL with several studies showing dramatic improvements in OS among those who received HAART [27–30]. The international prognostic index (IPI) which incorporates age, performance status, tumor state, serum LDH, and the number of sites of extranodal disease is a useful means of stratifying risk in aggressive lymphoma in both the non-HIV and HIV settings [31, 32]. In the HAART era, further refinements to prognostic factors have incorporated CD4+ cell counts to IPI as well as use of effective HAART. A multivariate analysis in patients with ARL showed that those with the most favorable IPI and a CD4+ count of >100 cells/*μ*L had a predicted 1-year survival of 82% compared to 15% in those with an unfavorable IPI and CD4+ count <100 cells/*μ*L [33]. Furthermore, in a retrospective study of 192 patients, the complete response rates and the median OS for ARL had improved to 57% and 43.2 months, respectively, in the HAART era compared to 32% and 8.3 months in the pre-HAART era [30]. The importance of HAART and CD4+ cell count was also underscored in a prospective study of 485 ARL patients undergoing risk-adaptive intensive chemotherapy [24]. A time-dependent covariates analysis showed that the significant factors for OS were HAART therapy, HIV score (based on performance status, prior AIDS diagnosis, and CD4+ count <100 cells/*μ*L), and the IPI [24]. More recently, a report by the Collaboration of observational HIV epidemiological research Europe (COHERE) study group showed that risk factors for death in ARL included low nadir CD4+ cell counts and a history of injection drug use [4]. 

## 4. Treatment of Newly Diagnosed ARL

ARLs are aggressive lymphomas and roughly 80% of patients present with advanced stage disease. Half of these patients will have gastrointestinal involvement and 30% will have bone marrow involvement [3, 14]. In addition, approximately 5 to 20 percent of patients with ARL have spread to the central nervous system (CNS) at the time of presentation, typically in the form of lymphomatous meningitis [34]. It is not clear that patients with HIV infection are inherently at higher risk for leptomeningeal involvement. It is more likely that the high incidence of CNS disease is related to the greater frequency of extranodal disease and Burkitt histology [35]. Lymphomatous meningitis may also present at the time of disease recurrence, particularly in patients at high risk for occult leptomeningeal involvement not treated with intrathecal prophylactic therapy during initial treatment of the lymphoma. 

Several prospective studies have reported using regimens such as cyclophosphamide, doxorubicin, vincristine, and prednisone (CHOP); methotrexate, bleomycin, doxorubicin, cyclophosphamide, vincristine, and dexamethasone (m-BACOD); infusional cyclophosphamide, doxorubicin, and etoposide [6, 7, 23, 36]. These studies have combined the high-grade DLBCL and Burkitt or Burkitt-like lymphoma subtypes with little difference in outcome among the various histologic subtypes. Patients who were most likely to achieve a complete remission with chemotherapy were those who had less disease burden, no bone marrow or CNS involvement, no prior AIDS-defining illness, and an adequate performance status. 

The introduction of HAART has led to dramatic improvement s in morbidity and mortality for PLWA. Such individuals now have an OS that is near or comparable to that of HIV seronegative individuals [21, 37]. 

The use of HAART has facilitated the use of standard-dose and even dose-intensive chemotherapy regimens with reasonable safety to patients with ARL. With use of HAART, leading to improvements in HIV viral suppression and patients' immune status, more recent studies have demonstrated that patients with ARL can be treated with standard-dose lymphoma protocols without experiencing undue toxicity [10, 23, 38, 39]. In addition, patients with ARL who receive chemotherapy now achieve a median OS comparable to the outcome in the non-immunosuppressed population [21, 23, 24, 40]. 

The United States National Cancer Institute (NCI)-sponsored AIDS Malignancy Clinical Trials Consortium (AMC) compared dose-reduced or full-dose cyclophosphamide, doxorubicin, vincristine, and prednisone (CHOP) with concomitant HAART with complete remission (CR) of 30% and 48% in the reduced dose and full-dose groups, respectively [33]. The German ARL study group also reported results of full-dose CHOP in patients with ARL stratified into standard- (0-1 factors) and high- (2-3 factors) risk based on CD4+ count <50 cells/*μ*L, prior OI and performance status ≥3. Standard-risk group patients had a CR of 79%, similar to that achieved in non-HIV infected patients with similar histologically aggressive lymphomas, while the CR was 29% in the high-risk group [32]. A more recent study by Groupe d'Etude des Lymphomes de l'Adulte (GELA) and Gruppo Italiano Cooperativo AIDS e Tumori (GICAT) reported on results of risk-adapted intensive chemotherapy in ARL patients [30]. A total of 485 patients were randomly assigned to chemotherapy after stratification according to “HIV score” based on performance status, prior AIDS diagnosis, and CD4+ count of <100 cells/*μ*L. A total of 218 good-risk patients (HIV score 0) received ACVBP (doxorubicin, cyclophosphamide, vindesine, bleomycin, and prednisolone) or CHOP; 177 intermediate-risk patients (HIV score 1) received CHOP or low-dose CHOP (Ld-CHOP); 90 poor-risk patients (HIV score 2-3) received Ld-CHOP or VS (vincristine and steroid). The 5-year OS in the good-risk group was 51% for ACVBP versus 47% for CHOP; in the intermediate-risk group, 28% for CHOP versus 24% for Ld-CHOP; in the poor-risk group, 11% for Ld-CHOP versus 3% for vs. The only significant factors for OS were HAART therapy, HIV score, and the IPI score, but not chemotherapy regimen, suggesting that in ARL patients, HIV score, IPI score, and HAART affect survival but not the dose intensity of the CHOP-based chemotherapy.

Infusional therapy may overcome some drug resistance and high tumor proliferation in aggressive lymphomas. A study of infusional dose-adjusted EPOCH (etoposide, prednisone, vincristine, cyclophosphamide, and doxorubicin) in 39 newly diagnosed ARL reported a CR in 74% of patients, and at a median followup of 53 months, disease-free survival (DFS), and OS of 92% and 60%, respectively [9]. An alternative infusional regimen which incorporated CDE (cyclophosphamide, doxorubicin, and etoposide) was employed by the US Eastern Cooperative Oncology Group (ECOG). Study (E1494) enrolled 98 patients with ARL. At two-year followup a CR of 45% was achieved and failure-free survival (FFS) and OS were 36% and 43%, respectively [7]. 

A number of recent clinical trials have further sought to combine anti-CD20 chimeric monoclonal antibody (rituximab) with ARL-based chemotherapy based on its superior efficacy when added to treatment of lymphoma in non-HIV infected patients. In a pooled analysis of three ARL prospective phase II studies evaluating rituximab in combination with infusional CDE, the CR was 70% and the estimated 2-year FFS and OS rates were 59% and 64%, respectively [39]. An increased risk of life-threatening infections was noted; 14% of patients were diagnosed with OIs during or within 3 months of completion of R-CDE, and 8% of patients died from infectious complications. In the multicenter AMC 010 phase III trial, 150 ARL patients were randomized at a ratio of 2 : 1 to receive CHOP and rituximab or CHOP only [10]. Results were provocative—with a median followup of 137 weeks there was a non-statistically significant trend towards improvement in time-to-progression (TTP: 125 weeks versus 85 weeks), progression-free survival (PFS: 45 weeks versus 38 weeks), and OS (139 weeks versus 110 weeks). Survival was significantly influenced by CD4+ count and IPI score. Although there was a trend towards a higher CR rate for those patients treated in the rituximab arm (58% versus 47%, *P* = 0.147), there were also substantially more deaths due to treatment-related infections in the rituximab arm (14% versus 2%, *P* = 0.027) with the majority of those deaths occurring in patients with CD4+ counts <50 cells/*μ*L at study entry. 

In contrast to AMC 010, a phase II study from France did not show an increase in the risk of life-threatening infections while treating newly diagnosed ARL patients with R-CHOP [10]. In this cohort of 61 patients, R-CHOP produced a CR of 77% and a 2-year OS of 75% with only 1 patient death attributed to infection. 

Rituximab has also been evaluated in conjunction with infusional EPOCH. In a randomized phase II AMC trial 034, rituximab was given either concurrently just before each infusional EPOCH chemotherapy cycle or sequentially (weekly for 6 weeks) after completion of all chemotherapy (4–6 cycles) in ARL [12]. CR was 73% in the concurrent arm, compared to 55% in the sequential arm. Toxicity was comparable in the 2 arms, although patients with a baseline CD4+ <50 cell/*μ*L had a high infectious death rate in the concurrent arm. The investigators concluded that concurrent rituximab and infusional EPOCH is an effective regimen for ARL that merits further evaluation, but that caution is needed for those patients with severe immunodeficiency. 

To summarize, rituximab has been shown to improve response rates and overall survival for patients with aggressive NHL who were HIV negative and treated with CHOP, without an increased risk of infectious complications. Rituximab, when given sequentially with chemotherapy, is associated with improved control of ARL; however, in a single clinical trial, the use of rituximab accounted for a higher risk of severe infection than has been seen for patients with NHL who were HIV negative. Several more recent ARL studies combining chemotherapy with rituximab have not, however, led to an increased risk of death due to infectious complications. 

Most oncologists favor administration of rituximab with chemotherapy, although caution should be exercised for using rituximab in severely immunosuppressed patients with CD4+ counts of less than 50 cells/*μ*L. Mature results from an ECOG study which randomizes non-HIV-infected patients with DLBCL to R-EPOCH or RCHOP will likely influence the way ARL is treated [41]. R-CHOP is more commonly offered in community settings because of ease of administration, because of physician familiarity with this regimen, and because no other regimen has, in a randomized and prospective study, outperformed R-CHOP for the treatment of DLBCL. However, oncologists who have substantial experience with infusional therapy may reasonably choose R-EPOCH based on favorable phase II data. 

Several ongoing clinical trials are evaluating novel chemotherapeutic combinations with biologic agents in this setting of ARL as listed in Table 2.

The appropriate timing of institution of HAART in relationship to multiagent chemotherapy has been evaluated in several clinical trials but with no clear answers. In a small study involving ARL patients, antiretroviral control was a significant factor in the ability to attain CR [38]. In a more recent trial evaluating safety and efficacy of liposomal doxorubicin when substituted for doxorubicin in the CHOP regimen effective HIV viral control during chemotherapy was associated with significantly improved survival, but CRs were attained independent of HIV viral control [23]. The AMC 010 study reported the feasibility of combining R-CHOP with concomitant HAART in 65 patients. Only one OI occurred during chemotherapy administration. Cyclophosphamide clearance was reduced compared with historical controls, without clinical significance. Additional studies of CHOP-based chemotherapy and HAART have yielded median survival periods of approximately 2 years [27, 29, 42]. For patients who continue HAART while receiving chemotherapy, CD4+ cell counts decline by 50%, but these values gradually improve and typically return to baseline within 6 months to 1 year of completing lymphoma treatment [43]. For patients who continue to receive effective HAART during chemotherapy, HIV viral replication tends to remain suppressed below the limits of detection using commercial-based polymerase chain reaction assays. 

Chemotherapy without concomitant antiretroviral therapy also has been studied in the HAART era. Reasons for HAART omission include concerns of drug interactions with chemotherapy and poor patient adherence because of nausea or vomiting. This could lead to heightened toxicity and the emergence of multidrug-resistant HIV quasispecies. In the NCI EPOCH study, HIV viral load increased and CD4+ cell counts decreased while patients received chemotherapy, but both parameters improved rapidly following the reintroduction of HAART at the completion of chemotherapy [12]. In the hands of experienced investigators, the temporary discontinuation of HAART for 4 to 6 months, while patients received chemotherapy did not lead to persistent and deleterious immunologic consequences. It is acceptable, therefore, to withhold HAART for a brief period of several months while patients receive ARL chemotherapy. In the absence of a head-to-head trial of administering or withholding HAART, both approaches are reasonable. HAART may, however, be particularly attractive to offer concurrently with chemotherapy for those patients who have a depleted CD4+ cell count at the time of ARL diagnosis, a history of opportunistic infections or other AIDS-related complications, appear likely to adhere to taking multiple oral medications, and for those patients who would likely achieve a nondetectable HIV viral load with HAART.

## 5. Treatment of Burkitt and Burkitt-Like NHL

Prospective studies of ARL generally have combined the high-grade DLBCL and Burkitt lymphoma/Burkitt-like lymphoma histologies, with little difference in outcome between the subtypes. In contrast, two retrospective reviews demonstrated substantially inferior outcomes for AIDS-associated Burkitts lymphoma in the HAART era in comparison with HIV-associated DLBCL when CHOP-like treatments were used [44]. Median survival was 5.7 months and 8 months for those with Burkitts lymphoma compared with 43.2 months and 22 months for those with DLBCL who received CHOP-like chemotherapy in the HAART era. Small retrospective and phase II studies of AIDS-associated Burkitt lymphoma reported the feasibility of using dose-intensive protocols with or without HAART treatment with response rates of approximately 70% [25, 45]. In addition, the NCI recently presented preliminary findings of abbreviated (three) cycles of dose-adjusted R-EPOCH in conjunction with prophylactic intrathecal CNS therapy for eight patients with AIDS-related Burkitt lymphoma [46]. All patients achieved a CR and remained in remission with a median followup of 4 years. These preliminary but encouraging results have led investigators to reconsider how best to treat HIV-infected patients with Burkitt lymphoma. AMC Study 048 recently completed accrual of patients with AIDS-related Burkitt lymphoma into a phase II study of high-dose, short-course chemotherapy regimen consisting of rituximab, cyclophosphamide, doxorubicin, vincristine, intrathecal cytarabine and methotrexate, ifosfamide, and etoposide (R-CODOX-M/IVAC). Final results from this study have yet to be reported. For patients with AIDS-related Burkitt lymphoma who have an adequate Karnofsky performance status an adequate CD4+ cell count and well-controlled HIV viremia, an intensive chemotherapy regimen similar to what is offered to patients with Burkitt lymphoma who are HIV negative is recommended.

## 6. Treatment of Relapsed ARL

Optimal chemotherapy for patients with relapsed/refractory ARL has not been defined and such patients are usually treated with regimens that are used for non-HIV-infected patients with relapsed/refractory aggressive lymphoma, such as ifosfamide, cisplatin and etopside (ICE), or etoposide, solumedrol, high-dose cytosine arabinoside, platinum (ESHAP) [47, 48]. These patients can also be considered for high-dose chemotherapy (HDCT) and peripheral autologous stem cell transplant (ASCT) if they have chemo-sensitive disease and have not exhausted HAART options. HAART therapy is feasible throughout the transplant process. In one report, 16 of 19 patients remained in remission after 2 years of followup [49]. Infectious complications were comparable with those that have been reported in the HIV-negative NHL patients who underwent ASCT and no long-term deterioration in immune function was noted. Several studies have reported success in the treatment of relapsed ARL using second-line therapy followed by HDCT and ASCT [50–52]. Among 68 ARL patients who underwent HDCT/ASCT from 20 institutions across Europe between the years 1999 and 2004, the PFS and OS were 56.5% and 61%, respectively, at a median followup of 32 months [53]. In a prospective study of HDCT/ASCT conducted by the GICAT group, 27 of the 50 enrolled patients underwent ASCT [54]. Intention-to-treat analysis showed a superior median OS for those who underwent ASCT (44 versus 33 months) compared to those who did not. Response to therapy significantly affected OS. In addition, AMC investigators used a preparative regimen of reduced-dose oral or intravenous busulfan in conjunction with cyclophosphamide as a preparative regimen for ASCT in patients with recurrent ARL and Hodgkin lymphoma [52]. The mean time to achievement of an absolute neutrophil count of greater than 0.5 × 10^9^/L was 11 days (range, 9 to 16 days). The median time to achievement of an unsupported platelet count of at least 20 × 10^9^/L was 13 days (range, 6 to 57 days). One patient died on day +33 after ASCT as a result of hepatic and renal occlusive disease and multiorgan failure. No other fatal regimen-related toxicity occurred and 10 (53%) of 19 evaluable patients were in complete remission at day +100. 

Recently, investigators from the City of Hope compared their experiences treating 29 consecutive patients with relapsed or progressive ARL who underwent ASCT to age, gender, histology, and stage-matched NHL controls who also underwent ASCT [55]. The median ARL patient age was 42 years and DLBCL/anaplastic large cell (16 patients) and Burkitt (11 patients) histologies predominated. The conditioning regimen most commonly consisted of CBV (cyclophosphamide 100 mg/kg, carmustine 450 mg/m^2^, and etoposide 60 mg/kg). The median time to engraftment was 10 days (range, 5–19 days). Treatment-related toxicities between the groups were comparable and 2-year overall survival at 75% was the same for both groups. 

European investigators have also reported results involving 53 HIV-positive and 53 HIV-negative matched lymphoma patients who underwent ASCT [20]. Incidence of PFS and OS were similar in both cohorts. The authors concluded that HIV-infected patients should be considered for ASCT according to the same criteria adopted for HIV-negative NHL patients. Furthermore, long-term followup data of ARL patients undergoing ASCT showed the nonrelapsed mortality (11% versus 4%, *P* = 0.18), 2-year DFS (76% versus 56%, *P* = 0.33) and the 2-year point estimates of OS (74% versus 75%, *P* = 0.93) were comparable to matched HIV-negative NHL controls [55]. These studies are preliminary but published experience with ASCT for ARL is accumulating [20, 52, 56–59]. Larger studies (some of which are currently underway, Table 3) with longer followup are needed to better define the optimal ARL conditioning regimen and to better delineate patient selection criteria. 

Gene therapy is an emerging technology that holds promise for patients with relapsed ARL. Recently, a stable expression of a lentiviral vector encoding anti-HIV RNAs in blood stem cells was transplanted into four such patients who were undergoing HDT followed by ASCT [60]. They each received gene-modified hematopoietic progenitor cells expressing 3 RNA-based anti-HIV moieties (tat/rev short hairpin RNA, TAR decoy, and CCR5 ribozyme). The gene-modified cells showed no differences in their hematopoietic potential compared with nontransduced cells; by day +11 all four patients were successfully engrafted with a persistent expression of the vector and the introduced small interfering RNA and ribozyme.

## 7. Supportive Care and Late-Treatment Complications

Attention to supportive care measures is essential for patients with ARL. Judicious use of hematopoietic stimulants including the newest formulation of pegylated granulocyte-colony stimulating factor (G-CSF) may help ameliorate chemotherapy-induced neutropenia. 

The US Food and Drug Administration (FDA) has issued a warning to medical providers that erythropoiesis-stimulating agents (darbepoetin alfa and epoetin alfa) are associated with a high rate of thrombosis for patients with cancer [61]. For these patients, the use of erythropoiesis-stimulating agents also may lead to a heightened risk of cancer progression and death [62]. Until these concerns are more fully evaluated, it is best to transfuse packed red blood cells when patients with ARL develop symptomatic anemia.

 To minimize cardiac toxicities, pegylated anthracyclines have been used for the treatment of ARL, and liposomal anthracyclines also may offer some of the pharmacokinetic benefits of infusional doxorubicin but without the need for patients to be connected to cumbersome infusion pumps for several days' duration. Single institution studies suggested that substituting liposomal anthracyclines for doxorubicin in multiagent lymphoma protocols was both safe and effective [23, 63]. AMC Study 047 substituted liposomal doxorubicin for doxorubicin in the R-CHOP regimen for patients with ARL [64]. However, a disappointing CR rate of only 37% was achieved. 

Infectious complications associated with ARL may be minimized by using prophylactic fluroquinolone antibiotics and azoles during periods of protracted neutropenia. All patients should receive Pneumocystitis *jiroveci* prophylaxis (e.g., dapsone, inhaled pentamidine, atovaquone) regardless of initial CD4+ cell count. Trimethoprim-Sulfamethoxazole is infrequently used because of its potential to exacerbate myelosuppresasion. 

With longer life expectancy following chemotherapy, late-treatment-related complications are emerging as a serious concern after treatment for ARL. Chemotherapy-related acute myelogenous leukemia (11q21 deletion with 2 additional copies of MLL gene at 10q) has been reported in an HIV-positive individual 48 months after therapy with R-EPOCH for ARL [65]. Similarly, three cases of secondary malignancy in the setting of ASCT have been reported [53]. It remains unknown whether the rate of secondary malignancies is intrinsically increased in the setting of HIV infection. 

## 8. Concluding Remarks

Epidemiologic reports clearly indicate that the incidence of ARL has decreased since the introduction of HAART among patients with AIDS. Chemo-immunotherapy for the most common subtype of ARL, DLBCL, is improving, with several phase II clinical trials showing good outcomes employing R-CHOP or R-EPOCH. CR rates for those patients with good prognosis features rival those of similarly matched non- HIV infected patients with DLBCL. Better supportive care, employing the use of G-CSF, prophylactic antibiotics, and HAART either during or shortly after completion of chemotherapy may ameliorate the increased toxicity of concurrent immunotherapy, thereby improving survival of patients with ARL. Clinical trials investigating the role of autologous and allogeneic HSCT in relapsed/refractory ARL are currently underway. With effective treatments and longer life expectancy following chemotherapy, late-treatment-related complications are emerging, and how best to mitigate and manage these complications is an emerging challenge.

## Figures and Tables

**Table 1:** Who Classification of lymphoid malignancies associated with HIV infection.

*Lymphomas also occurring in immunocompetent patients*	
Burkitt and Burkitt-like lymphomas	
Diffuse large B-cell lymphomas	
Centroblastic	
Immunoblastic (including primary CNS lymphoma)	
Extranodal MALT lymphoma (rare)	
Peripheral T-cell lymphoma (rare)	
Classical Hodgkin lymphoma	

*Lymphoma occurring more specifically in HIV-positive patients*	
Primary effusion lymphoma	
Plasmablastic lymphoma of the oral cavity	

*Lymphoma occurring in other immunodeficiency states*	
Polymorphic B-cell lymphoma (PTLD-like) (rare)	

MALT: marginal zone lymphoma of mucosa-associated lymphoid tissue; PTLD: posttransplant lymphoproliferative disorder; CNS: central nervous system.

*Source: *[13].

**Table 2:** Active clinical trial protocols evaluating chemotherapy in ARL.

Study identifier	Phase	Study regimen	Start date	Primary endpoint
NCT00006436	II	EPOCH and Rituximab in ARL	October 2000	PFS
NCT00598169 (AMC 053)	I/II	Bortezomib, Ifosfamide, Carboplatin, and Etoposide ± Rituximab in relapsed ARL	November 2007	ORR and safety
NCT01092182	II	Dose-adjusted R-EPOCH in Burkitt or c-MYC+ DLBCL	February 2010	PFS, EFS, and OS
NCT01193842 (AMC 075)	I/II	R-CHOP or R-EPOCH ± Vorinostat in AIDS-related DLBCL	October 2010	ORR, MTD, and toxicity

EPOCH: etoposide, prednisone, vincristine, cyclophosphamide, and doxorubicin; ARL: AIDS-related lymphoma; PFS: progression-free survival; AMC: AIDS Malignancy Consortium; ASCT: autologous stem cell transplant; PET: positron emitting tomography; CT: computed tomography; ABVD: doxorubicin, bleomycin, vinblastine, dacarbazine; BEACOPP: bleomycin, etoposide, doxorubicin, cyclophosphamide, vincristine, procarbazine, prednisone; HL: Hodgkin's lymphoma; OS: overall survival; ORR: overall response rate; DLBCL: diffuse large B cell lymphoma; EFS: event-free survival; R-CHOP: rituximab, cyclophosphamide, doxorubicin, vincristine, prednisone; MTD: maximum tolerated dose.

*Source: http://clinicaltrials.gov/.*

**Table 3:** Active clinical trial protocols evaluating hematopoietic stem cell transplant in ARL.

Study identifier	Phase	Study	Start date	Primary endpoint
NCT00345865	II	Cyclophosphamide + TBI versus carmustine, cyclophosphamide and etoposide conditioning followed by ASCT in AIDS-related NHL or HL	November 2005	DFS and OS
NCT00641381	II	Carmustine, Etoposide, Cyclophosphamide and ASCT in ARL	March 2000	Feasibility and toxicity
NCT00858793	I/II	HDT and transplantation of gene-modified ASCT for high-risk ARL	October 2008	Adverse events
NCT00968630	II	Immune response after HSCT in HIV-positive patients with hematologic cancer	August 2009	HIV-specific immune response
NCT01045889	II	R-CHOP followed by HDT and ASCT	January 2007	OS
NCT01141712 (AMC 071)	II	HDT (BEAM) and ASCT in ARL	February 2011	OS
NCT01410344	II	Allogeneic HSCT for hematological cancers and myelodysplastic syndromes in HIV-infected individuals	September 2011	NRM

TBI: total body irradiation; NHL: non-Hodgkin's lymphoma; HL: Hodgkin's lymphoma; DFS: disease-free survival; OS: overall survival; HDT: high-dose therapy; ASCT: autologous stem cell transplant; HSCT: hematopoietic stem cell transplant; R-CHOP: rituximab, cyclophosphamide, doxorubicin, vincristine, prednisone; BEAM: carmustine, etoposide, cytarabine, melphalan; NRM: nonrelapse mortality.

*Source: http://clinicaltrials.gov/.*

## Abbreviations

AIDS: Acquired immunodeficiency syndrome
HAART: Highly active antiretroviral therapy
ARL: AIDS-related lymphoma
HIV: Human immunodeficiency virus.
